# TDP43 pathology in the brain, spinal cord, and dorsal root ganglia of a patient with FOSMN

**DOI:** 10.1212/WNL.0000000000007008

**Published:** 2019-02-26

**Authors:** Alexander M. Rossor, Zane Jaunmuktane, Martin N. Rossor, Glen Hoti, Mary M. Reilly

**Affiliations:** From the MRC Centre for Neuromuscular Diseases (A.M.R., M.M.R.), Department of Neurodegenerative Disease (Z.J.), Queen Square Brain Bank (Z.J., G.H.), and Dementia Research Centre (M.N.R.), UCL Institute of Neurology; National Hospital for Neurology and Neurosurgery (A.M.R., M.M.R., M.N.R.); and Division of Neuropathology (Z.J.), National Hospital for Neurology and Neurosurgery, University College London Hospitals NHS Foundation Trust, Queen Square, UK.

## Abstract

**Objective:**

To describe the histopathologic features of a case of facial-onset sensory and motor neuronopathy (FOSMN).

**Methods:**

We describe a postmortem examination performed on a 54-year-old man with FOSMN associated with personality change.

**Results:**

Postmortem examination revealed TAR DNA-binding protein (TDP) 43 proteinopathy with widespread distribution. TDP43 pathology was seen in the neurons and glial cells and was most pronounced in the subthalamic nucleus followed by the spinal cord, including dorsal root ganglia, brainstem, and other deep cerebral nuclei. In the medial temporal lobe, neocortex and subcortical hemispheric white matter TDP43 pathologic inclusions were very rare. In contrast to TDP43 pathologies associated with typical amyotrophic lateral sclerosis (ALS) or frontotemporal dementia (FTD)–TDP, in this case, there were more frequent TDP43-positive oligodendroglial, coiled body–like cytoplasmic inclusions than neuronal inclusions. Neuronal cytoplasmic TDP43 inclusions with globular and skein-like morphology were seen in both anterior horn cells and dorsal root ganglia. No β-amyloid, α-synuclein, or significant hyperphosphorylated tau pathology was seen.

**Conclusion:**

This case provides further evidence that FOSMN is a neurodegenerative disease characterized by TDP43 pathology. Despite minimal cortical TDP43 pathology, the clinical features of the behavioral variant of FTD in this patient suggest that FOSMN may fall within or overlap with the FTD-ALS spectrum.

Facial-onset sensory and motor neuronopathy (FOSMN) is a rare clinical syndrome characterized by asymmetric facial numbness or paresthesia, bulbar palsy, and facial weakness, which may progress to the upper limbs.^1^ The FOSMN syndrome was first described in 2006, and since then, >40 cases have been described.^2,3^ Onset is typically in the fifth to seventh decade (but has been reported in patients as young as 7 years of age^4^), and the rate of progression can vary from months to decades.

The pathogenesis of FOSMN remains controversial. Initial reports described the presence of anti-ganglioside antibodies and response to immunotherapy,^5^ whereas others have described a progressive and terminal decline, resistant to immunotherapy and suggestive of bulbar-onset amyotrophic lateral sclerosis (ALS).^3,6–8^ Two of 3 postmortem studies of patients with FOSMN have revealed the presence of TAR DNA-binding protein (TDP) 43 inclusions in the brainstem nuclei and cervical motor neurons.^3,7,8^ In this article, we describe a patient with FOSMN and behavioral change in whom postmortem examination revealed frequent pathologic TDP43 inclusions in the deep cerebral nuclei, brainstem, and spinal cord and rarely in the medial temporal lobe, frontal cortex, and dorsal root ganglia (DRG).

## Methods

Formalin-fixed, paraffin-embedded brain and spinal cord tissue was available from selected regions (table 1). The paraffin sections were cut at 5 μm, mounted on glass slides, and stained with routine hematoxylin and eosin. Representative 5-μm sections were immunostained for TDP43, β-amyloid, α-synuclein, hyperphosphorylated tau, p62, and CD68 with the following antibodies: TDP43 (2E2-D3, 1:3,000, Abcam, Cambridge, UK), β-amyloid (6F3D, 1:50, DAKO, Glostrup, Denmark), α-synuclein (KM51, 1:50, Leica/Novocastra, Buffalo Grove, IL), AT8, (MN1020, 1:100, Invitrogen, Carlsbad, CA), p62 (3/P62LCK Ligand, 1:100, BD Transduction, East Rutherford, NJ), and CD68 (PG-M1, 1:100, DAKO), respectively. Immunostaining was performed on either a BondMax autostainer (Leica Microsystems, Wetzlar, Germany) or a Roche (Basel, Switzerland) Ventana Discovery automated staining platform following the manufacturer's guidelines, using biotinylated secondary antibodies and a horseradish peroxidase–conjugated streptavidin complex and diaminobenzidine as a chromogen. All immunostainings were carried with appropriate controls. Gliosis, microglial activity, and the density of TDP43 pathologic inclusions were scored semiquantitatively.

**Table 1:**
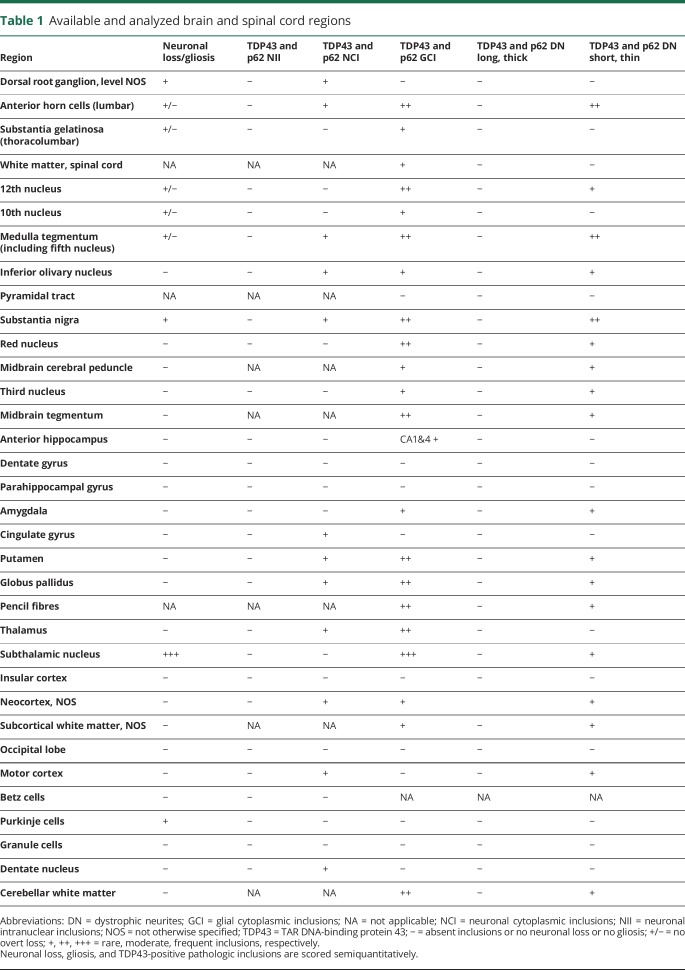
Available and analyzed brain and spinal cord regions

### Data availability statement

All data relevant to this study are contained within the manuscript.

## Results

A 54-year-old right-handed man presented with symptoms of facial numbness, dysarthria, and dysphagia and inappropriate behavior. Nine years previously, after a dental extraction, he developed numbness of the left upper lip that over 5 years spread to involve his tongue and left cheek and was associated with dysphagia and nasal regurgitation. A collateral history obtained from his wife reported that since the onset of the dysphagia, the patient's behavior had changed and he had difficulty maintaining his train of thought. There was no family history of neurodegenerative disease.

A Mini-Mental State Examination was normal; however, he was noted to have an inappropriate jocular manner. Clinical examination revealed a cachectic man. There was diminished light touch sensation in all territories of the trigeminal nerve and diminished pinprick sensation in the left mandibular territory. The corneal reflexes were absent. There was bilateral facial myokymia. The gag reflex was absent, and there was weakness of neck flexion, along with wasting and fasciculation of the tongue. In the upper limbs, there was wasting and weakness of the deltoids. There was no wasting, weakness, or sensory disturbance of the lower limbs. The knee and ankle reflexes were preserved, and the plantar responses were absent.

Nerve conduction studies performed 5 years after the onset of his symptoms revealed normal sensory and motor action potentials and conduction velocities in the upper and lower limbs (table 2). EMG demonstrated large individual motor units firing at high rates in a grossly reduced interference pattern, indicative of chronic denervation, in the sternocleidomastoid and orbicularis oculi. There was no evidence of acute denervation. Blink reflexes were absent bilaterally.

**Table 2:**
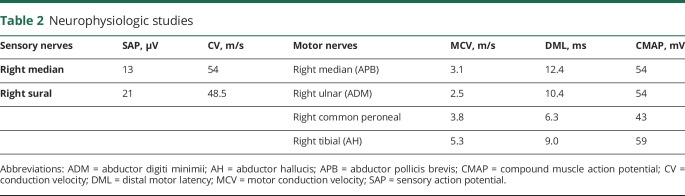
Neurophysiologic studies

MRI scans of the brain and spine were normal. Fluorodeoxyglucose PET CT scan revealed an enlarged jugular lymph node that was subsequently biopsied and showed nonspecific reactive lymphoid hyperplasia. Duodenal biopsies were negative for Whipple disease. A lip biopsy was unremarkable. CSF examination was normal, including oligoclonal bands and Whipple PCR. Genetic testing for Kennedy disease, dentatorubral-pallidoluysian atrophy, and spinocerebellar ataxia types 1, 2, 3, 6, and 7 was negative. Routine blood tests were all normal.

Formal neuropsychometry demonstrated a verbal IQ of 115 and performance IQ of 114, with average subtest scores except for arithmetic and picture completion, which were superior. Recognition memory tests were almost flawless (words 48 of 50, faces 50 of 50). Although he performed adequately on tests sensitive to frontal lobe dysfunction, he was noted to be inappropriately jocular during testing.

The patient was diagnosed with a progressive brainstem syndrome. In hindsight, the clinical syndrome is FOSMN, but this diagnosis had not been described at the time the patient presented. Because an inflammatory brainstem disorder remained a possibility, the patient was treated with a 3-month course of oral steroids, but his condition continued to deteriorate, and he was readmitted to hospital with an aspiration pneumonia. A percutaneous gastrostomy tube was inserted, but against medical advice, he continued to eat and drink, leading to a final episode of aspiration pneumonia, of which he died, 9 years after the onset of his symptoms.

### Postmortem examination

Pathologic TDP43-positive inclusions were seen in the form of neuronal cytoplasmic inclusions (NCIs), oligodendroglial, coiled body–like cytoplasmic inclusions (GCIs), and dystrophic neurites (figure). GCIs and dystrophic neurites were more frequent than NCIs. TDP43 pathology was most prominent in the subthalamic nucleus, followed by the thalamus, putamen, and globus pallidus (other deep cerebral nuclei were not available for the assessment), brainstem, and spinal cord (table 1). NCIs with globular and skein-shaped morphology were seen in both motor (anterior horn cells) and sensory (dorsal root ganglion) neurons. In the neocortex, subcortical hemispheric white matter, anterior hippocampus, amygdala, and cingulate gyrus, TDP43 pathology was present but very rare. In the cerebellum, there were GCIs in the white matter and rare NCIs in the dentate nucleus but none in the cerebellar cortex (table 1). TDP43-positive inclusions also showed immunoreactivity for p62. There were no diagnostically specific p62 immunoreactive inclusions in the cerebellum or hippocampal dentate gyrus to suggest *C9ORF72* pathology or neuronal intranuclear inclusions characteristic of *VCP* or *p62* mutations. Microglial activity, highlighted with CD68 immunostaining, was most prominent in the subthalamic nucleus with comparably mild activation elsewhere, including the corticospinal tracts and anterior and posterior nerve roots in the spinal cord. No β-amyloid, α-synuclein, or significant hyperphosphorylated tau pathology was seen.

**Figure:**
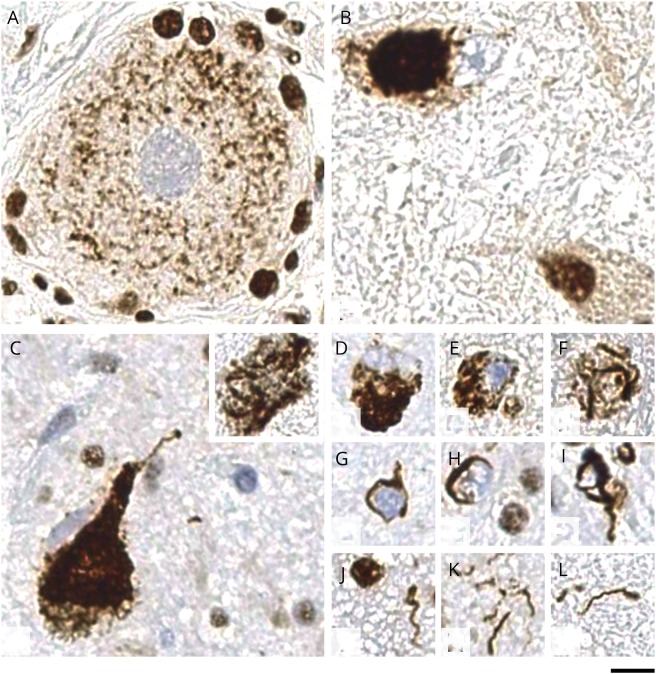
TDP43 proteinopathy in FOSMN (A) Pathologic thread-like deposits in a neuron within dorsal root ganglion. (B) Globular cytoplasmic inclusion in the lumbar anterior horn motor neuron. (C) Large globular and skein-like (inset) cytoplasmic inclusions in pigmented neurons of the substantia nigra. (D–F) Various morphologies of neuronal cytoplasmic inclusions. (G– I) glial cytoplasmic inclusions, all of which show similar, coiled body–like morphology. (J–L) Appearances of dystrophic neurites, all of which are short and curved with no evidence of long neurites. All sections are immunostained with nonphosphorylated TAR DNA-binding protein 43 (TDP43) antibody, which detects normal nuclear TDP43 labeling and shows absent nuclear labeling in cells where there is TDP43 mislocalization from the nucleus to cytoplasm or cell processes. Scale bar: 10 μm in panels A–L. FOSMN = facial-onset sensory and motor neuronopathy.

## Discussion

In this report, we describe a case of FOSMN with personality change and a postmortem examination characterized by TDP43-positive inclusions in the cerebral cortex, brainstem, and spinal cord motor neurons and DRGs. Our findings corroborate the findings of 2 of 3 previous postmortem examinations of patients with FOSMN in which TDP43 inclusions were identified in the brainstem.^3,7,8^ This report provides further evidence that FOSMN may be considered a *forme fruste* of bulbar-onset ALS, a notion further supported by the behavioral change and TDP43 inclusions in the frontal cortex. We were unable to measure β-amyloid_42_ and tau in the CSF because this assay was not available 15 year ago at the time of the patient's illness. It should also be noted that although the presence of TDP43 inclusions in the neocortex was unequivocal, they were infrequent.

The clinical syndrome of FOSMN was first described in 2006, several years after our patient’s death.^2^ Further histologic analysis was prompted by reports of TDP43 inclusions in postmortem tissue of patients with FOSMN.^8^ At the time of our patient's illness, mutations in *C9ORF72*, *FUS*, and *TARDBP* had not yet been discovered to cause ALS.

This report also describes the identification of TDP43 inclusions in the DRG of a patient with a sensory neuronopathy. This is an intriguing observation, and it is tempting to speculate that a neurodegenerative pathology, characterized by TDP43 inclusion in the DRG, may underlie a proportion of patchy asymmetric sensory neuronopathies that follow a progressive course and are resistant to immunosuppressive therapy.^9^ In further support of the pathogenicity of the TDP43 inclusions in the DRG is the recent report of a patient with an asymmetric patchy and progressive sensory and motor neuronopathy in whom a p.Arg382Pro missense mutation in *TARDBP* was discovered.^10^ This mutation is not present in the Exac database and has previously been described in patients with ALS.^11^ An additional distinctive feature seen in this patient and reported in the literature^8^ is the presence of widespread glial TDP43 pathology. TDP43-positive glial inclusions are also observed in both ALS and frontotemporal dementia–TDP, but to a much lesser extent, suggesting that there might be differences in molecular pathogenesis between FOSMN and ALS–frontotemporal dementia spectrum.

We describe TDP43 proteinopathy in a patient with FOSMN and personality change. The presence of TDP43 proteinopathy in the brain, spinal cord, and DRG and the discovery of a pathogenic *TDP-43* mutation in a patient with an asymmetric sensory and motor neuronopathy indicate that some sensory neuronopathies have a degenerative pathogenesis.

## Glossary

ALS: amyotrophic lateral sclerosis
DRG: dorsal root ganglia
FOSMN: facial-onset sensory and motor neuronopathy
GCI: oligodendroglial coiled body–like cytoplasmic inclusion
NCI: neuronal cytoplasmic inclusion
TDP: TAR DNA-binding protein
